# A human tissue‐specific transcriptomic analysis reveals a complex relationship between aging, cancer, and cellular senescence

**DOI:** 10.1111/acel.13041

**Published:** 2019-09-27

**Authors:** Kasit Chatsirisupachai, Daniel Palmer, Susana Ferreira, João Pedro de Magalhães

**Affiliations:** ^1^ Integrative Genomics of Ageing Group Institute of Ageing and Chronic Disease University of Liverpool Liverpool UK

**Keywords:** cell division, geriatric oncology, oncogenesis, transcriptome, tumor

## Abstract

Aging is the biggest risk factor for cancer, but the mechanisms linking these two processes remain unclear. Using GTEx and TCGA data, we compared genes differentially expressed with age and genes differentially expressed in cancer among nine human tissues. In most tissues, aging and cancer gene expression pattern changed in the opposite direction. The exception was thyroid and uterus, where we found transcriptomic changes in the same direction in aging and in their corresponding cancers. The overlapping sets between genes differentially expressed with age and genes differentially expressed in cancer across tissues were enriched for several processes, mainly cell cycle and the immune system. Moreover, cellular senescence signatures, derived from a meta‐analysis, changed in the same direction as aging in human tissues and in the opposite direction of cancer signatures. Therefore, transcriptomic changes in aging and in cellular senescence might relate to a decrease in cell proliferation, while cancer transcriptomic changes shift toward enhanced cell division. Our results highlight the complex relationship between aging and cancer and suggest that, while in general aging processes might be opposite to cancer, the transcriptomic links between human aging and cancer are tissue‐specific.

1

Aging is the biggest risk factor for cancer (de Magalhaes, 2013). However, the biological mechanisms behind this link are still unclear. Gene expression analyses have been used to study cancer (Cieslik & Chinnaiyan, 2018) and aging (de Magalhaes, Curado, & Church, 2009; Yang et al., 2015), but only a few studies have investigated the relationship between gene expression changes in these two processes (Aramillo Irizar et al., 2018). In particular, comparisons between human tissue‐specific genes differentially expressed with age (age‐DEGs) and genes differentially expressed in cancer (cancer‐DEGs) are lacking. Cellular senescence is a state of irreversible cell cycle arrest and has been regarded as an anti‐tumor mechanism (Campisi, 2013). However, accumulating evidence has suggested that senescent cells could also promote cancer (Demaria et al., 2017). Many studies have attempted to identify gene expression signatures of senescence (Kim et al., 2013), but comparisons between cellular senescence signatures, age‐DEGs, and cancer‐DEGs are also lacking.

The Genotype‐Tissue Expression (GTEx) Project has profiled gene expression from noncancerous tissues of nearly 1,000 individuals (age 20–79 years) over 53 sampled sites (Ardlie et al., 2015). The Cancer Genome Atlas (TCGA) has sequenced tumor samples from more than 10,000 patients covering 33 cancer types (Ding et al., 2018). Here, we investigated the relationship between transcriptomic changes in aged human tissue and their corresponding cancer by analyzing gene expression data from GTEx and TCGA. In addition, we conducted a meta‐analysis using publicly available datasets to identify cellular senescence signature genes and compared them with age‐DEGs and cancer‐DEGs.

We first identified age‐DEGs in 26 tissues from GTEx (v7), nine tissues (breast, colon, esophagus, liver, lung, prostate, stomach, thyroid, and uterus) were selected for subsequent analyses (Table S1). The numbers of significant age‐DEGs (*p*‐value with Benjamini–Hochberg correction < .05 and absolute fold change > 1.5; moderated *t* test) varied between different tissues (Figure 1a, Figure S1, Data S1). We identified cancer‐DEGs by analyzing nine TCGA datasets for which the tissues of origin were matched to the GTEx tissues used in this study (Table S1, Figure S2). The numbers of cancer‐DEGs (*p*‐value with Benjamini–Hochberg correction < .01 and absolute fold change > 2; moderated *t* test) are shown in Figure 1b (Data S2).

**Figure 1:**
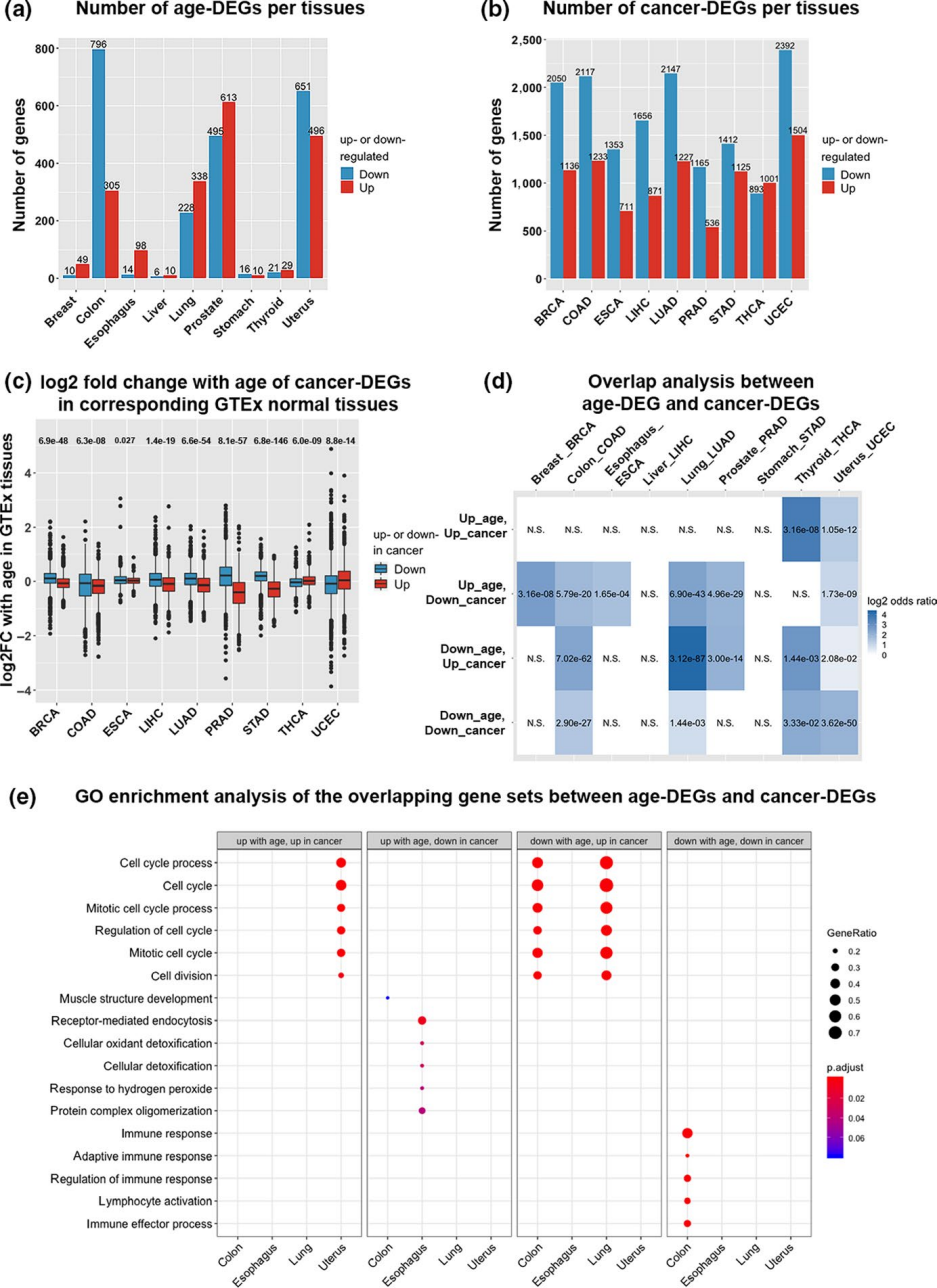
The relationship between age‐DEGs and cancer‐DEGs. (a) Number of age‐DEGs. (b) Number of cancer‐DEGs. The full study name of TCGA projects can be found in the Table S1. (c) Fold change with age in GTEx data of cancer‐DEGs. Numbers indicate *p*‐values. (d) Overlap between age‐DEGs and cancer‐DEGs. Numbers represent *p*‐values with Benjamini–Hochberg correction. N.S. denotes nonsignificant overlap. Colors correspond to odds ratio. (e) GO enrichment analysis of significantly overlapping gene sets. The plot shows examples of significant enriched terms (*p*‐value with Benjamini–Hochberg correction < .1)

After obtaining a list of cancer‐DEGs, we examined the fold change with age in GTEx tissues of the cancer‐DEGs. We observed a significantly (*p*‐value < .05; Mann–Whitney *U* test) higher fold change with age in genes down‐regulated with cancer when compared to genes up‐regulated with cancer for most cancer types, with the opposite being observed in two tissues: THCA‐thyroid and UCEC‐uterus (Figure 1c). We overlapped age‐DEGs and cancer‐DEGs for each tissue. Consistent with the result in Figure 1c, genes changing in the opposite direction between aging and cancer significantly overlapped more often than genes changing in the same direction in breast, colon, esophagus, lung, and prostate (Fisher's exact test, Benjamini–Hochberg correction) (Figure 1d). For thyroid, the overlap was significant for genes changing in the same direction. There was no significant overlap in liver and stomach, which might be explained by the small number of age‐DEGs in these tissues. In uterus, however, the overlap was significant in all cases. The same analyses were also performed for the brain and glioblastoma multiforme (GBM), where we found the same direction of transcriptomic changes between aging and cancer. However, due to the small number of control brain samples (five samples) in TCGA and because they lack information of the patient age, we decided to include the brain result only in the (Figure S3, Data S3).

We performed GO enrichment analysis and found that 6 out of 20 significantly overlapping sets were enriched in GO terms (Figure 1e, Data S3). Genes down‐regulated with age—up‐regulated in cancer, in colon, and lung, were related to cell cycle. Cell cycle terms were also enriched in genes up‐regulated with age—up‐regulated in cancer in the uterus. Uncontrolled cell proliferation in the aging uterus often leads to endothelial hyperplasia and could lead to endometrial cancer (Damle et al., 2013). Immune‐related terms were enriched in genes down‐regulated with age—down‐regulated in cancer in the colon. The immune system plays an important role in preventing cancer through immunosurveillance (Ribatti, 2017). Compromised immune function with age could provide an immunosuppressive microenvironment, allowing cancer cells to evade immunosurveillance (Fulop et al., 2010).

Our results highlight the tissue‐specific nature of transcriptomic changes in human aging and cancer, even though in general they changed in the opposite direction for most tissues, in line with another study (Aramillo Irizar et al., 2018). One possible interpretation is that molecular changes during aging processes may oppose cancer development. Another potential interpretation for our results, from an evolutionary perspective, is that aging‐related changes in tissue microenvironment, leading to the decrease in tissue robustness, might provide a selective advantage for cells harboring oncogenic mutations (Henry, Marusyk, Zaberezhnyy, Adane, & DeGregori, 2010; Parikh, Shuck, Gagea, Shen, & Donehower, 2018). We note that the incidences of thyroid and uterine cancer are different from others, they plateau at an younger age than other cancers (de Magalhaes, 2013). Interestingly, these are the organs in which we found the same direction of transcriptomic changes between aging and cancer. One limitation of our work is that we are unable to distinguish changes in transcriptome during aging within each cell type in the tissue or changes in tissue cell composition.

We next performed a meta‐analysis of 20 replicative senescence microarray datasets from the Gene Expression Omnibus (GEO) and identified 526 and 734 consistently over‐ and underexpressed genes (Table S2, Data S4). GO and KEGG enrichment analyses indicated that underexpressed signatures were related to cell cycle and DNA repair, while the overexpressed signatures were linked to immune processes and the p53 signaling pathway, a well‐established senescence pathway (Figure S4, Data S5).

Underexpressed senescence signatures overlapped with genes down‐regulated with age in the colon and lung, while overexpressed senescence signatures overlapped with genes up‐regulated with age in colon, lung, prostate, and thyroid (Fisher's exact test, Benjamini–Hochberg correction) (Figure 2a,b). Only the uterus displayed opposite results, in line with the up‐regulation in the aging uterus of cell cycle‐related genes (Figure 2a,b). For cancer genes, an opposite trend between cancer‐DEGs and senescence signatures emerged (Figure 2c,d), consistent with the anti‐cancer role of senescence. Only in thyroid cancer (THCA) the genes up‐regulated in cancer significantly overlapped with overexpressed senescence genes.

**Figure 2:**
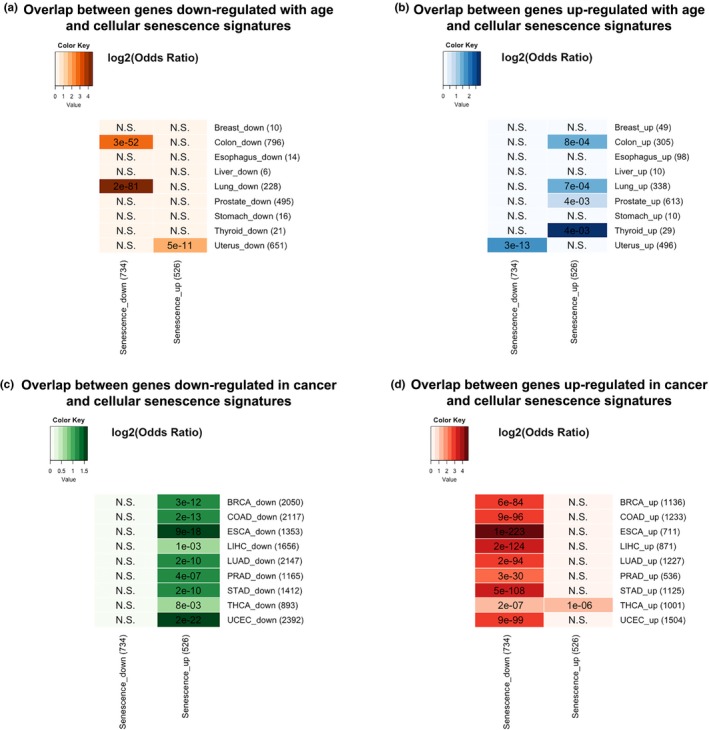
Overlap between cellular senescence signature genes and genes (a) down‐regulated with age, (b) up‐regulated with age, (c) down‐regulated in cancer, and (d) up‐regulated in cancer. Numbers represent *p*‐values with Benjamini–Hochberg correction. N.S. denotes nonsignificant overlap. Colors correspond to log2 odds ratio

In conclusion, our study shows that signatures of cellular senescence are activated in aged human tissues and our results further highlight the tissue‐specific relationship between aging and cancer. While in the majority of tissues gene expression aging changes are opposite of those in the corresponding cancers, thyroid and uterus showed similar changes. The overlapping genes between cancer and aging were linked to different processes, mainly cell cycle. This study provides novel insights into the complex relationship between transcriptomic changes in human aging, cancer, and cellular senescence.

## CONFLICT OF INTEREST

None declared.
