# Supplementary material for: A human tissue‐specific transcriptomic analysis reveals a complex relationship between aging, cancer, and cellular senescence
Source: Aging Cell. 2019 Sep 27;18(6):e13041. doi: 10.1111/acel.13041 (PMC6826163; doi:10.1111/acel.13041)
Supplement: Supplementary file 1 [file ACEL-18-e13041-s001.pdf]

Chatsirisupachai et al., ***A Human Tissue-Specific Transcriptomic Analysis Reveals a Complex Relationship between Ageing, Cancer, and Cellular Senescence***

Supporting Information

Methods

Tissue-specific genes differentially expressed with age (age-DEGs) from GTEx

The RNA-Seq based gene expression data of non-cancerous tissues (v7, January 2015 release) were download from the GTEx portal (https://gtexportal.org) (Consortium, 2015). RNA-Seq was performed using the Illumina TrueSeq library construction protocol. Reads were aligned to the human reference genome hg19/GRCh37 based on the GENCODE v19 and were processed as described in https://gtexportal.org/home/documentationPage. Out of 30 tissues provided by GTEx, four tissues (bladder, cervix uteri, fallopian tube, and kidney) with low samples (11, 11, 7, and 45, respectively) were excluded from the analysis. For each tissue, we identified differentially expressed genes with age using the following linear regression model:

$$Y_{ij} = \alpha Age_i + \beta Sex_i + \gamma Death_i + \varepsilon_{ij}$$

Where Y_{ij} is the expression level of gene j in sample i, Age_i denotes the age of sample i. Sex_i denotes the sex of sample I, $Death_i$ denotes the death classification of sample i based on the 4-point Hardy scale (Ferreira *et al.*, 2018), and ε_{ij} denotes the error term. It should be noted that dataset downloaded from GTEx portal did not provide the actual age of each sample, the age ranges, i.e. 20-29, 30-39, 40-49, 50-59, 60-69 and 70-79, were provided instead. We then approximated the age of each sample to 25, 35, 45, 55, 65 and 75, respectively. Because our main aim was to compare the age-DEGs with cancer-DEGs from TCGA, and there was a large difference between the number of the total genes in GTEx (56,202) and TCGA (20,532), we focus only on protein coding genes. Protein-coding genes were identified using the R package *biomaRt* (version 2.36.1) (Durinck *et al.*, 2009), based on Ensembl release 92 (April 2018). After removing the non-coding genes, there were 18,851 protein-coding genes. To remove the low expressed genes, genes with expression less than 1 count per million (cpm) in more than 30 percent of samples were excluded. Raw read counts were normalized using TMM normalization and were voom transformed to remove heteroscedasticity from the

count data. The linear model for each gene was generated by using the *limma* package in R (version 3.36.5) (Ritchie *et al.*, 2015). Genes were considered to be significant differentially expressed genes with age if the empirical Bayes moderated t-statistics and their associated adjust *P*-value (Benjamini-Hochberg method) < 0.05 and absolute fold change across 50 years of age (from 25 to 75 years old) > 1.5. The genes in GTEx were in ensemble id, we then converted them into entrez id format using *biomaRt*. Lists of age-DEGs from all 26 tissues are provided in the **Data S1**. Age distributions of the samples from GTEx were shown in **Figure S1**.

We selected nine tissues for further analysis, including breast, colon, esophagus, liver, lung, prostate, stomach, thyroid and uterus. This selection was based on the criteria that 1) each of these tissues had a matched TCGA project from the same tissue of origin and 2) the number of solid normal samples in those TCGA projects were more than 10. We also performed the analyses for the brain. However, the number of solid normal samples in TCGA-GBM project was only 5 and they lack information of patient age, we included the brain-GBM results in supplementary figures instead. Numbers of samples used in our study were summarized in **Table S1**. Most of the age-DEGs were tissue-specific, indicating the tissue specificity of age-DEGs, consistent with a previous report (Yang *et al.*, 2015).

Genes differentially expressed in cancer (cancer-DEGs) from 10 TCGA projects

The TCGA level 3 RNA-Seq based gene expression data for 10 TCGA projects used in our analysis (BRCA (Cancer Genome Atlas, 2012b), COAD (Cancer Genome Atlas, 2012a), ESCA (Cancer Genome Atlas Research *et al.*, 2017), GBM (Brennan *et al.*, 2013), LIHC (Cancer Genome Atlas Research Network. Electronic address & Cancer Genome Atlas Research, 2017), LUAD (Cancer Genome Atlas Research, 2014b), PRAD (Cancer Genome Atlas Research, 2015), STAD (Cancer Genome Atlas Research, 2014a), THCA (Cancer Genome Atlas Research, 2014c), and UCEC (Cancer Genome Atlas Research *et al.*, 2013)) were download from FireBrowse (http://firebrowse.org) in June 2019. RNA sequencing was performed on the Illumina Hi-Seq platform and aligned to the human reference genome GRCh37. The data then processed using RNASeqV2 pipeline of TCGA, which provide the RSEM expected counts. Protein-coding genes were identified using the *biomaRt* R package (version 2.36.1). After removing non-coding genes, there were 18,548 protein-coding genes included in subsequent analysis. To remove the genes with low expression, genes with

expression less than 1 count per million (cpm) in more than 30 percent of samples were excluded. The *limma* package in R was used to identify genes significantly differentially expressed between cancer and normal samples. The moderated t statistic *P*-value after correction by Benjamini-Hochberg method < 0.01 with an absolute fold-change > 2 were used to determine the statistical significance. Lists of cancer-DEGs from all 10 TCGA projects were provided in the supplementary **Data S2**. Age distributions of primary tumour and solid normal samples were shown in **Figure S2**.

Fold change with age in normal tissues of cancer-DEGs

We examined the fold change with age of the cancer-DEGs. After obtaining the cancer-DEGs from TCGA, we identified the fold change with age in normal tissues in GTEx of these cancer-DEGs. Although not every cancer-DEG was found in the GTEx data from the corresponding tissue because lowly expressed genes had been filtered out before performing linear regression, the majority of them retained (**Data S6**). For each tissue, the fold change with age in normal tissue of up-regulated genes in cancer and down-regulated genes in cancer were compared using Mann-Whitney *U* test.

Meta-analysis to identify cellular senescence signature genes

To identify cellular senescence signature genes, the meta-analysis of 20 cellular senescence GEO datasets was performed. The datasets included in the analysis were shown in **Table S2**. We first separately identified differentially expressed genes in each dataset. The probe annotation for each dataset was slightly different, depended on the platform. In general, the steps are 1) Download the dataset from GEO, 2) Probe annotation to match probe ID with entrez ID using either the dataset's own GPL platform files, R packages (*AnnotationDbi* version 1.44.0 and *org.Hs.eg.db* version 3.7.0), or bioDBnet (https://biodbnet-abcc.ncifcrf.gov/db/db2db.php) (Mudunuri *et al.,* 2009) 3) Remove probes which do not match to any entrez ID 4) Remove probes which match to multiple entrez ID 5) Average intensity of probes which match to the same entrez ID. The differential expression analysis was performed using limma. Genes with a *P*-value below 0.05 and absolute fold change > 1.5 were considered putatively differentially expressed genes. For the datasets with only one non-senescent sample and one senescent sample, only fold change cut-off was used to determine differentially expressed genes. There are two platforms (GPL2937 and GPL2947) in the dataset GSE3460, so we separately analysed each platform and treated them as two different datasets in the meta-analysis. After that, the results from all datasets were then combined

using a binomial distribution and the false discovery rate set at $Q < 0.05$ using the same method as (de Magalhaes *et al.,* 2009). In total, there were 1,259 senescence signature genes, with 526 overexpressed and 734 underexpressed. We found that 442 of 1,259 senescence signature genes were significantly overlapped with the signature of replicative senescence provided by Hernandez-Segura et al. (Hernandez-Segura *et al.,* 2017) (*P*-value = 2.9e-161; Fisher's exact test), indicating the reliability of our data.

Overlap analysis

The overlap analyses were performed using the *GeneOverlap* package in R (version 1.16.0). In each experiment, background was set differently depending on where the genes come from. For the overlap between age-DEGs from GTEx and cancer-DEGs from TCGA, all protein coding genes in GTEx data (18,851 genes) were used as a background. For the overlap between age-DEGs and cellular senescence signature genes, all protein coding genes in combined datasets used for the meta-analysis (18,878 genes) were used as a background. For the overlap between cancer-DEGs and cellular senescence signature genes, all protein coding genes in combined datasets used for the meta-analysis (18,878 genes). The overlap was considered significant if an adjusted *P*-value < 0.05 (Fisher's exact test followed by Benjamini-Hochberg correction).

Functional enrichment analysis

Gene ontology (GO) enrichment analysis was performed using the *clusterProfiler* package in R (version 3.8.1) (Yu *et al.,* 2012). A GO term was considered to be an enriched term if an adjusted *P*-value < 0.1 (Benjamini-Hochberg correction). The union set of age-DEGs and cancer-DEGs in each of the four conditions for each tissue was employed as a background for GO enrichment test of the overlapping gene set in that tissue. For example, the union set of genes up-regulated with age in colon and genes up-regulated in COAD was used as a background for GO enrichment analysis of overlapping genes between genes up-regulated with age in colon and genes up-regulated in COAD. KEGG pathway enrichment analysis was performed on the underexpressed and overexpressed cellular senescence signatures. A pathway was enriched if an adjusted *P*-value < 0.05 (Benjamini-Hochberg correction). All protein coding genes in combined datasets used for the meta-analysis (18,878 genes) were employed as a background.

Validating the results with recount2 data

Because GTEx and TCGA had processed the data differently, we confirmed that this has not affected the results of the comparison between age-DEGs and cancer-DEGs by using data from recount2 (Collado-Torres *et al.,* 2017). Recount2 is a project to standardize the pipeline of analysis and provide the ready-to-analyze data, thus TCGA and GTEx (version 6) data have been processed using the same pipeline and they contain the same number of genes (58037 genes in total, including 19732 protein-coding genes). We performed the analyses following the same methods as describe above with this recount2 data and confirmed that age-DEGs and cancer-DEGs change in the same direction in thyroid, uterus, and brain, while change toward the opposite direction in the other tissues (**Figure S5**). We, however, keep the results analysed from the GTEx database (version 7) and TCGA downloaded from FireBrowse, because GTEx (version 6) contain less samples, particularly in the oldest age group (70 – 79 years old).

List of Supplementary Tables, Supplementary Figures and Additional Data

- Table S1 – Number of GTEx and TCGA samples used in the study
- Table S2 – GEO datasets for meta-analysis of cellular senescence signatures
- Figure S1 – Age distribution of samples from 26 GTEx tissues
- Figure S2 – Age distribution of primary tumour and normal tissues from 10 TCGA projects used in this study
- Figure S3 – The relationship between age-DEGs and cancer-DEGs in brain-GBM
- Figure S4 – GO and KEGG pathway enrichment analysis of cellular senescence signatures
- Figure S5 – Confirmation of the comparison between age-DEGs and cancer-DEGs using recount2 data
- Data S1 – List of age-DEGs in GTEx tissues
- Data S2 – List of cancer-DEGs in nine TCGA projects
- Data S3 – List of enriched terms from GO enrichment analysis of overlap genes between age-DEGs and cancer-DEGs
- Data S4 – List of cellular senescence signature genes
- Data S5 – GO and KEGG pathway enrichment analysis of cellular senescence signature genes
- Data S6 – Lists and fold change with age of cancer-DEGs presented in GTEx data

Supplementary Table S1 – Number of GTEx and TCGA samples included in this study

**GTEx Tissues**		**TCGA Projects**			
				Samples	
Tissue Name	Cases	Disease Name	Project	Primary Solid Tumour	Solid Tissue Normal
Brain	1671	Glioblastoma Multiforme	GBM	153	5
Breast	290	Breast invasive carcinoma	BRCA	1093	112
Colon	506	Colon adenocarcinoma	COAD	285	41
Esophagus	1018	Esophageal carcinoma	ESCA	184	11
Liver	174	Liver hepatocellular carcinoma	LIHC	371	50
Lung	425	Lung adenocarcinoma	LUAD	515	59
Prostate	152	Prostate adenocarcinoma	PRAD	497	52
Stomach	261	Stomach adenocarcinoma	STAD	415	35
Thyroid	444	Thyroid carcinoma	THCA	501	59
Uterus	110	Uterine Corpus Endometrial Carcinoma	UCEC	186	24

Supplementary Table S2 – GEO datasets for meta-analysis of cellular senescence signatures

GEO	Platform ID	References	Tissue	Cell Type/ Cell Line	Proliferating Cells	Senescent Cells
GSE17077	GPL1352	(Gruber *et al.*, 2010)	Annulus	-	8	8
GSE13330	GPL570	(Pazolli *et al.*, 2009)	Foreskin	BJ Fibroblasts	6	6
GSE49860	GPL5639	(Imai *et al.*, 2014)	-	Diploid Fibroblasts	1	1
GSE41714	GPL10558	(Kim *et al.*, 2013)	Dermal	Diploid Fibroblasts	2 (2 days)	2 (30 and >30 days)
GSE37091	GPL6480	(Jong *et al.*, 2013)	Umbilical vein	Endothelial cell (HUVECS)	2	2
GSE54095	GPL6244	(Guerrero *et al.*, 2015)	Coronary Artery	Endothelial Cells	4	4
GSE3460	GPL2937	(Schwarze *et al.*, 2002)	Prostate	Epithelial Cells	2	2
GSE3460	GPL2947	(Schwarze *et al.*, 2002)	Prostate	Epithelial Cells	1	1
GSE3731	GPL2990	(Zhang *et al.*, 2003)	Mammary	Epithelial Cells (HMEC) (48R and 184)	8	8
GSE36640	GPL570	(Shah *et al.*, 2013)	-	Fibroblast/IMR90	5	5
GSE19018	GPL570	-	-	Fibroblast/IMR90	3	3
GSE28863	GPL5175	(Cao *et al.*, 2011)	-	Fibroblasts	12	12
GSE3730	GPL2990	(Zhang *et al.*, 2003)	-	Fibroblasts (WS1, WI38 and BJ)	13	13
GSE56530	GPL570	(Medeiros Tavares Marques *et al.*, 2017)	Umbilical Cord Vein	Mesenchymal Stem Cells	9	9
GSE35957	GPL570	(Benisch *et al.*, 2012)	Bone Marrow	Mesenchymal Stem Cells	5	5
GSE46019	GPL6244	(Frobel *et al.*, 2014)	Bone Marrow	Mesenchymal Stromal Cells	3	3
GSE15919	GPL1528	(Binet *et al.*, 2009)	-	MRC5 Fribroblasts	6	6
GSE10570	GPL4133	(Bhatia *et al.*,	Prostate	NHP8	3	3

		2008)				
GSE6762	GPL4693	(Johung *et al.*, 2007)	-	Primary Fibroblasts	4	4
GSE687	GPL506	(Hardy *et al.*, 2005)	Mammary Stroma	Primary Fibroblasts	12	12
GSE34303	GPL4133	(Ren *et al.*, 2013)	Bone Marrow	Stromal Cells	20	20

Figure S1 – Age distribution of samples from 26 GTEx tissues

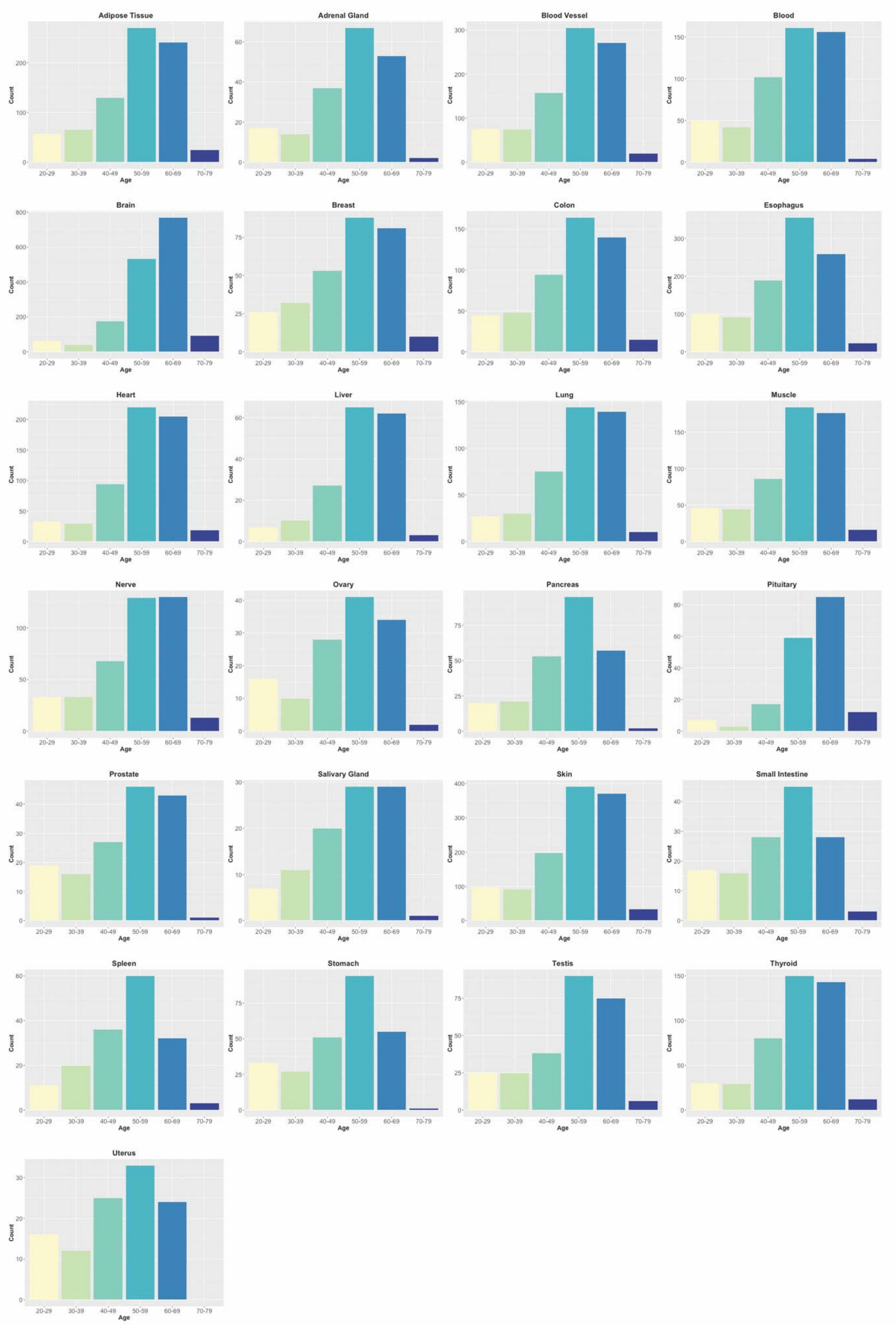

Figure S2 – Age distribution of primary tumour and normal tissues from 10 TCGA projects used in this study

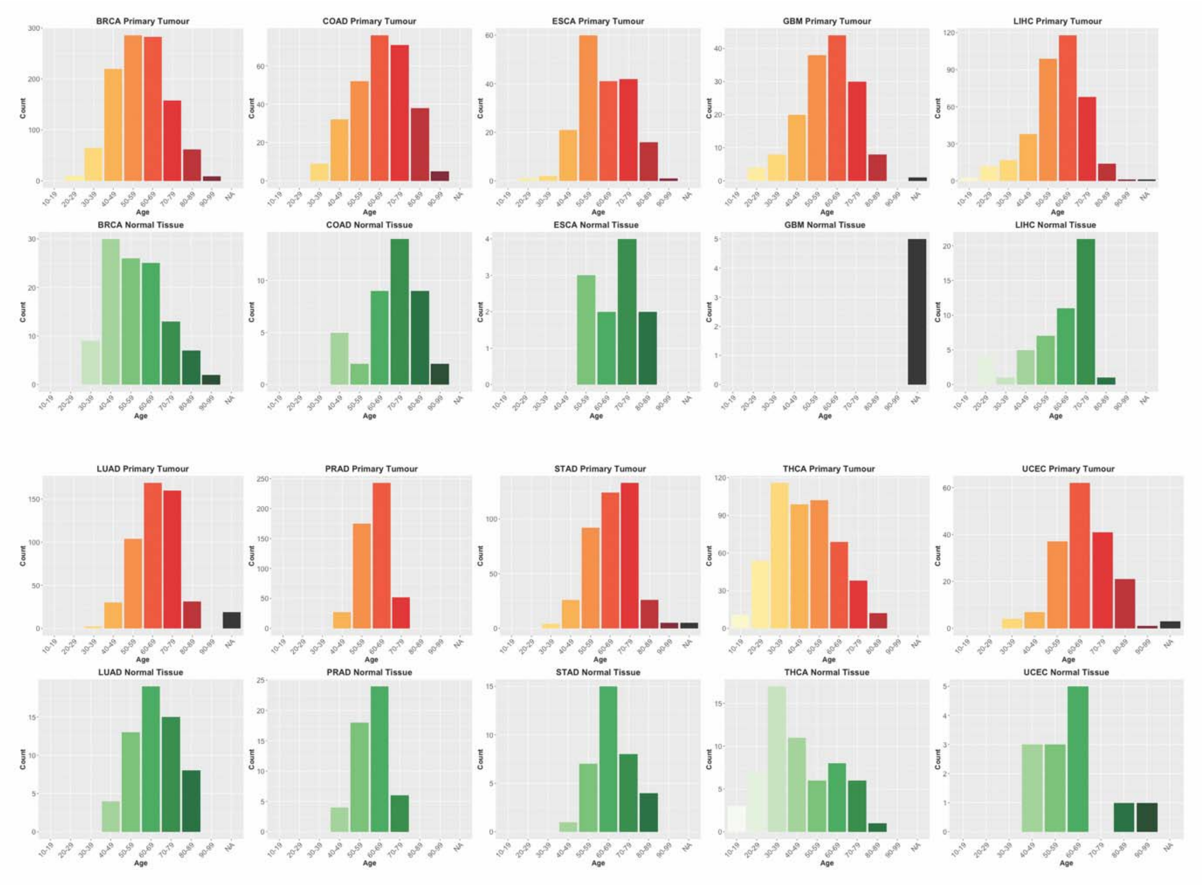

Figure S3 – The relationship between age-DEGs and cancer-DEGs in brain-GBM

(a) Fold change with age in GTEx brain data of cancer-DEGs from GBM. A number indicates *P*-values (Mann-Whitney *U* test). (b) Overlap between age-DEGs and cancer-DEGs from brain. Numbers represent FDR (Fisher's exact test, Benjamini-Hochberg correction). N.S. denotes non-significant overlap. Colours of the heatmap correspond to odds ratio. (c) GO enrichment analysis of significant overlap gene sets. The plot shows examples of significant enriched terms (FDR < 0.1). The full list of enriched terms was in the Data S3.

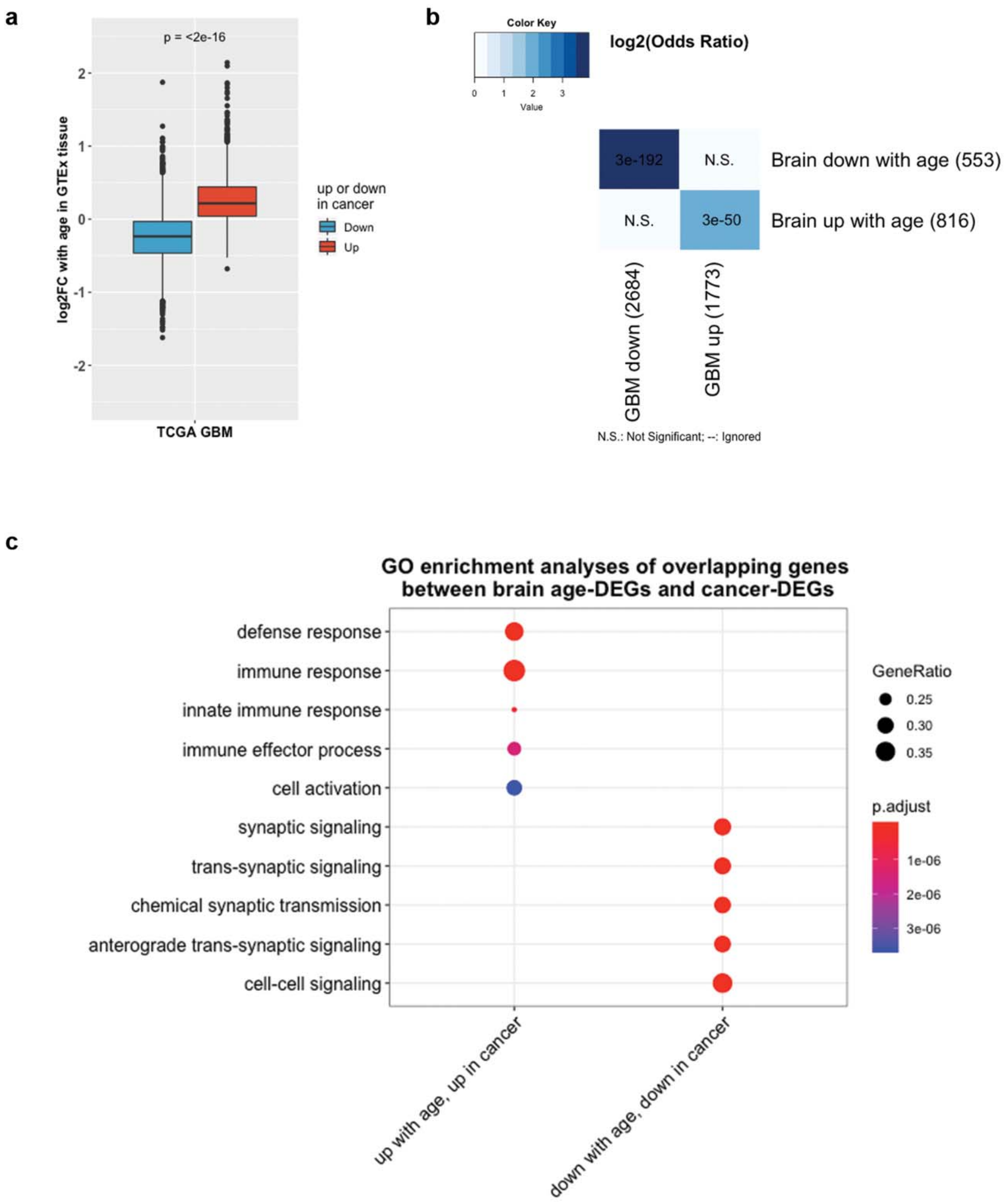

Figure S4 – GO and KEGG pathway enrichment analysis of cellular senescence signatures

(a) GO and (b) KEGG pathway enrichment analysis of significant overlap gene sets. The plot shows examples of significant enriched terms (FDR < 0.05). The full list of enriched terms was in the Data S5.

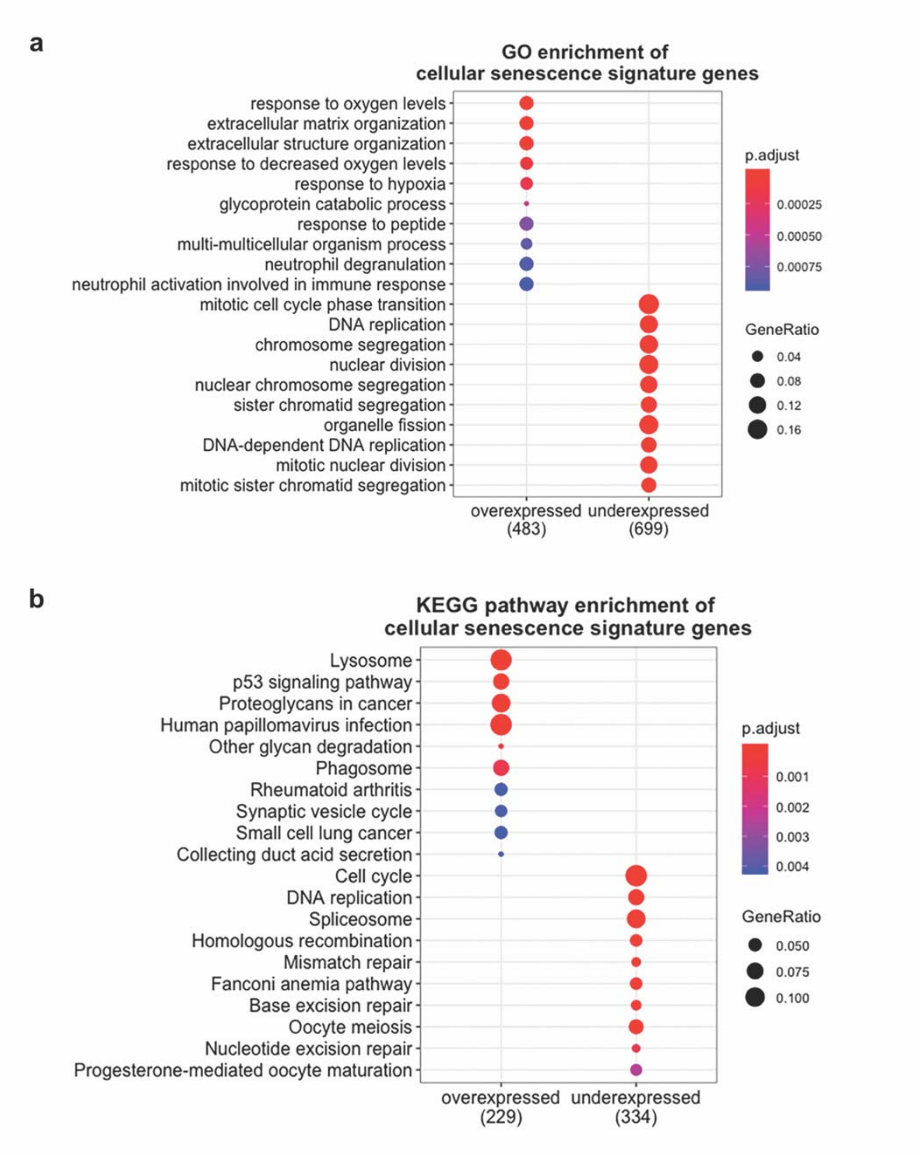

Figure S5 – Confirmation of the comparison between age-DEGs and cancer-DEGs using recount2 data

Numbers of (a) age-DEGs from GTEx (b) cancer-DEGs from TCGA, RNA-Seq data obtained from recount2 resource. (c) Fold change with age in GTEx data of cancer-DEGs (Compare to Figure 1c and Figure S3a). (d) Overlap analysis between tissue-specific age-DEGs and cancer-DEGs (Compare to Figure 1d and Figure S3b). Because only one age-DEG identified from stomach, the overlap analysis between age-DEG from stomach and cancer-DEGs from STAD was not able to performed. (e) GO enrichment analysis of significant overlap gene sets (Compare to Figure 1e and Figure S3c). The plot shows examples of significant enriched terms (P-values with Benjamini-Hochberg correction < 0.1).

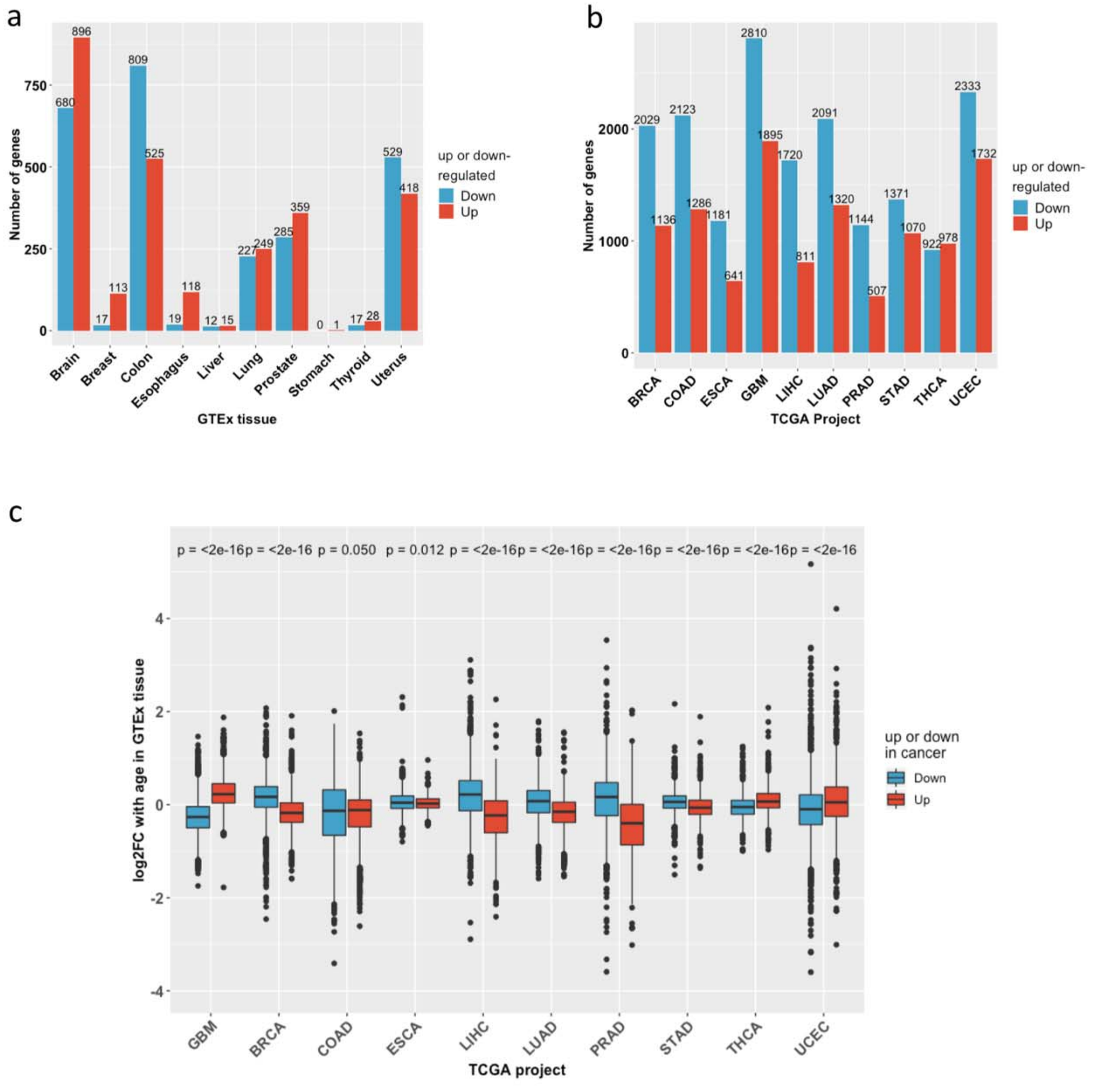

d

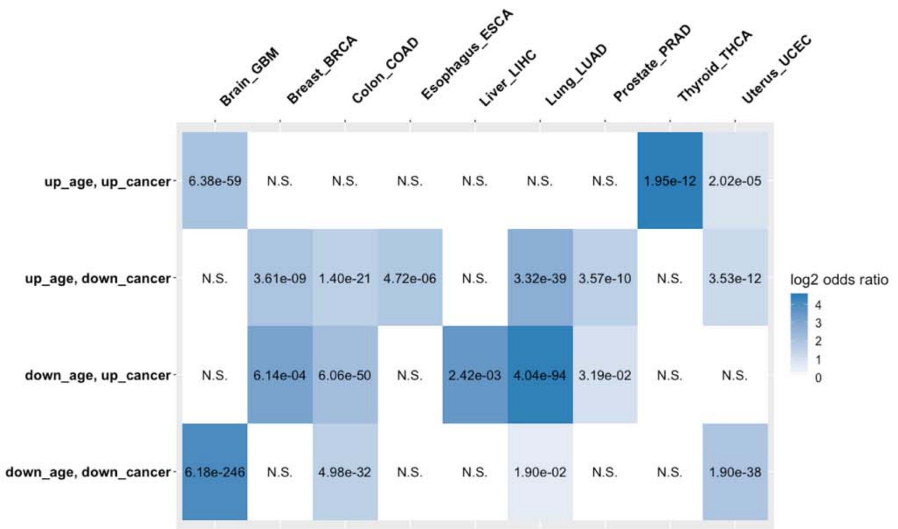
Brain_GBM
Breast_BRCA
Colon_COAD
Esophagus_ESCA
Liver_LIHC
Lung_LUAD
Prostate_PRAD
Thyroid_THCA
Uterus_UCEC
up_age, up_cancer
up_age, down_cancer
down_age, up_cancer
down_age, down_cancer
6.38e-59 N.S. N.S. N.S. N.S. N.S. N.S. 1.95e-12 2.02e-05
N.S. 3.61e-09 1.40e-21 4.72e-06 N.S. 3.32e-39 3.57e-10 N.S. 3.53e-12
N.S. 6.14e-04 6.06e-50 N.S. 2.42e-03 4.04e-94 3.19e-02 N.S. N.S.
6.18e-246 N.S. 4.98e-32 N.S. N.S. 1.90e-02 N.S. N.S. 1.90e-38
log2 odds ratio
4
3
2
1
0

e

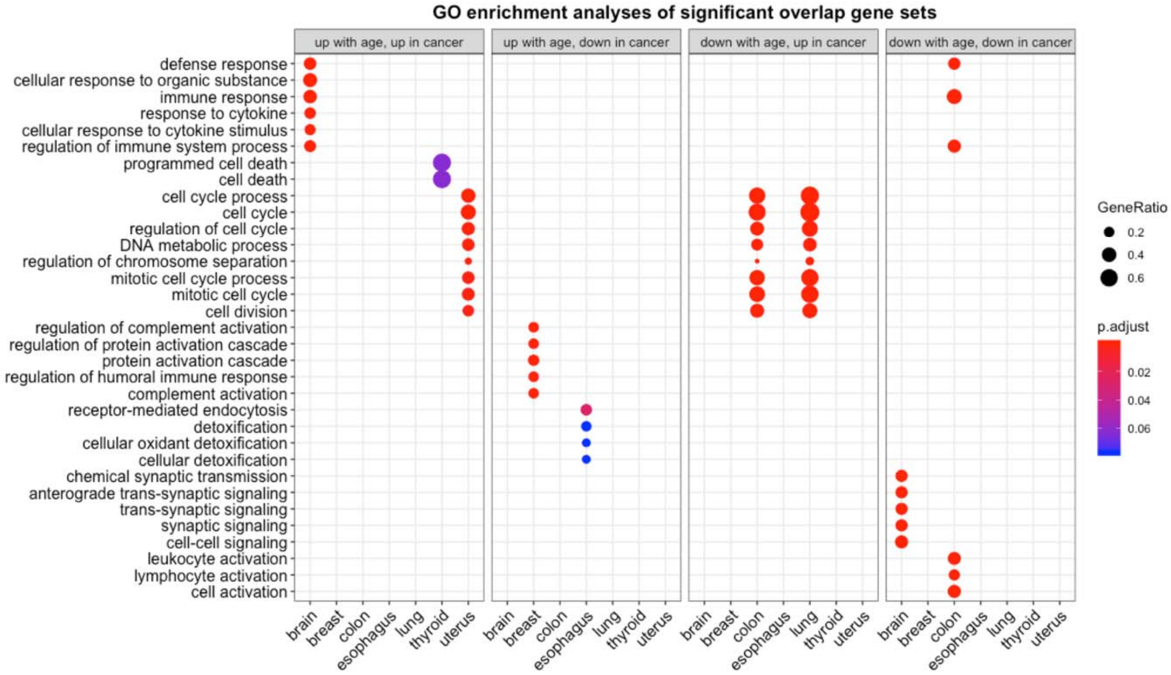
GO enrichment analyses of significant overlap gene sets
up with age, up in cancer
up with age, down in cancer
down with age, up in cancer
down with age, down in cancer
defense response
cellular response to organic substance
immune response
response to cytokine
cellular response to cytokine stimulus
regulation of immune system process
programmed cell death
cell death
cell cycle process
cell cycle
regulation of cell cycle
DNA metabolic process
regulation of chromosome separation
mitotic cell cycle process
mitotic cell cycle
cell division
regulation of complement activation
regulation of protein activation cascade
protein activation cascade
regulation of humoral immune response
complement activation
receptor-mediated endocytosis
detoxification
cellular oxidant detoxification
cellular detoxification
chemical synaptic transmission
anterograde trans-synaptic signaling
trans-synaptic signaling
synaptic signaling
cell-cell signaling
leukocyte activation
lymphocyte activation
cell activation
brain
breast
colon
esophagus
lung
thyroid
uterus
GeneRatio
0.2
0.4
0.6
p.adjust
0.02
0.04
0.06

References

Benisch, P., Schilling, T., Klein-Hitpass, L., Frey, S. P., Seefried, L., Raaijmakers, N., ... Jakob, F. (2012). The transcriptional profile of mesenchymal stem cell populations in primary osteoporosis is distinct and shows overexpression of osteogenic inhibitors. *PLoS One*. **7**, e45142.

Bhatia, B., Jiang, M., Suraneni, M., Patrawala, L., Badeaux, M., Schneider-Broussard, R., ... Tang, D. G. (2008). Critical and distinct roles of p16 and telomerase in regulating the proliferative life span of normal human prostate epithelial progenitor cells. *J Biol Chem*. **283**, 27957-27972.

Binet, R., Ythier, D., Robles, A. I., Collado, M., Larrieu, D., Fonti, C., ... Pedeux, R. (2009). WNT16B is a new marker of cellular senescence that regulates p53 activity and the phosphoinositide 3-kinase/AKT pathway. *Cancer Res*. **69**, 9183-9191.

Brennan, C. W., Verhaak, R. G., McKenna, A., Campos, B., Noushmehr, H., Salama, S. R., ... Network, T. R. (2013). The somatic genomic landscape of glioblastoma. *Cell*. **155**, 462-477.

Cancer Genome Atlas, N. (2012a). Comprehensive molecular characterization of human colon and rectal cancer. *Nature*. **487**, 330-337.

Cancer Genome Atlas, N. (2012b). Comprehensive molecular portraits of human breast tumours. *Nature*. **490**, 61-70.

Cancer Genome Atlas Research, N. (2014a). Comprehensive molecular characterization of gastric adenocarcinoma. *Nature*. **513**, 202-209.

Cancer Genome Atlas Research, N. (2014b). Comprehensive molecular profiling of lung adenocarcinoma. *Nature*. **511**, 543-550.

Cancer Genome Atlas Research, N. (2014c). Integrated genomic characterization of papillary thyroid carcinoma. *Cell*. **159**, 676-690.

Cancer Genome Atlas Research, N. (2015). The Molecular Taxonomy of Primary Prostate Cancer. *Cell*. **163**, 1011-1025.

Cancer Genome Atlas Research, N., Analysis Working Group: Asan, U., Agency, B. C. C., Brigham, Women's, H., Broad, I., ... Project Team: National Institutes of, H. (2017). Integrated genomic characterization of oesophageal carcinoma. *Nature*. **541**, 169-175.

Cancer Genome Atlas Research, N., Kandoth, C., Schultz, N., Cherniack, A. D., Akbani, R., Liu, Y., ... Levine, D. A. (2013). Integrated genomic characterization of endometrial carcinoma. *Nature*. **497**, 67-73.

Cancer Genome Atlas Research Network. Electronic address, w. b. e., & Cancer Genome Atlas Research, N. (2017). Comprehensive and Integrative Genomic Characterization of Hepatocellular Carcinoma. *Cell*. **169**, 1327-1341 e1323.

Cao, K., Blair, C. D., Faddah, D. A., Kieckhaefer, J. E., Olive, M., Erdos, M. R., ... Collins, F. S. (2011). Progerin and telomere dysfunction collaborate to trigger cellular senescence in normal human fibroblasts. *J Clin Invest*. **121**, 2833-2844.

Collado-Torres, L., Nellore, A., Kammers, K., Ellis, S. E., Taub, M. A., Hansen, K. D., ... Leek, J. T. (2017). Reproducible RNA-seq analysis using recount2. *Nat Biotechnol*. **35**, 319-321.

Consortium, G. T. (2015). Human genomics. The Genotype-Tissue Expression (GTEx) pilot analysis: multitissue gene regulation in humans. *Science*. **348**, 648-660.

de Magalhaes, J. P., Curado, J., & Church, G. M. (2009). Meta-analysis of age-related gene expression profiles identifies common signatures of aging. *Bioinformatics*. **25**, 875-881.

Durinck, S., Spellman, P. T., Birney, E., & Huber, W. (2009). Mapping identifiers for the integration of genomic datasets with the R/Bioconductor package biomaRt. *Nat Protoc*. **4**, 1184-1191.

Ferreira, P. G., Munoz-Aguirre, M., Reverter, F., Sa Godinho, C. P., Sousa, A., Amadoz, A., ... Guigo, R. (2018). The effects of death and post-mortem cold ischemia on human tissue transcriptomes. *Nat Commun*. **9**, 490.

Frobel, J., Hemeda, H., Lenz, M., Abagnale, G., Joussen, S., Denecke, B., ... Wagner, W. (2014). Epigenetic rejuvenation of mesenchymal stromal cells derived from induced pluripotent stem cells. *Stem Cell Reports*. **3**, 414-422.

Gruber, H. E., Hoelscher, G. L., Ingram, J. A., Zinchenko, N., & Hanley, E. N., Jr. (2010). Senescent vs. non-senescent cells in the human annulus in vivo: cell harvest with laser capture microdissection and gene expression studies with microarray analysis. *BMC Biotechnol*. **10**, 5.

Guerrero, A., Iglesias, C., Raguz, S., Floridia, E., Gil, J., Pombo, C. M., & Zalvide, J. (2015). The cerebral cavernous malformation 3 gene is necessary for senescence induction. *Aging Cell*. **14**, 274-283.

Hardy, K., Mansfield, L., Mackay, A., Benvenuti, S., Ismail, S., Arora, P., ... Jat, P. S. (2005). Transcriptional networks and cellular senescence in human mammary fibroblasts. *Mol Biol Cell*. **16**, 943-953.

Hernandez-Segura, A., de Jong, T. V., Melov, S., Guryev, V., Campisi, J., & Demaria, M. (2017). Unmasking Transcriptional Heterogeneity in Senescent Cells. *Curr Biol*. **27**, 2652-2660 e2654.

Imai, Y., Takahashi, A., Hanyu, A., Hori, S., Sato, S., Naka, K., ... Hara, E. (2014). Crosstalk between the Rb pathway and AKT signaling forms a quiescence-senescence switch. *Cell Rep*. **7**, 194-207.

Johung, K., Goodwin, E. C., & DiMaio, D. (2007). Human papillomavirus E7 repression in cervical carcinoma cells initiates a transcriptional cascade driven by the retinoblastoma family, resulting in senescence. *J Virol*. **81**, 2102-2116.

Jong, H. L., Mustafa, M. R., Vanhoutte, P. M., AbuBakar, S., & Wong, P. F. (2013). MicroRNA 299-3p modulates replicative senescence in endothelial cells. *Physiol Genomics*. **45**, 256-267.

Kim, Y. M., Byun, H. O., Jee, B. A., Cho, H., Seo, Y. H., Kim, Y. S., ... Yoon, G. (2013). Implications of time-series gene expression profiles of replicative senescence. *Aging Cell*. **12**, 622-634.

Medeiros Tavares Marques, J. C., Cornelio, D. A., Nogueira Silbiger, V., Ducati Luchessi, A., de Souza, S., & Batistuzzo de Medeiros, S. R. (2017). Identification of new genes associated to senescent and tumorigenic phenotypes in mesenchymal stem cells. *Sci Rep*. **7**, 17837.

Mudunuri, U., Che, A., Yi, M., & Stephens, R. M. (2009). bioDBnet: the biological database network. *Bioinformatics*. **25**, 555-556.

Pazolli, E., Luo, X., Brehm, S., Carbery, K., Chung, J. J., Prior, J. L., ... Stewart, S. A. (2009). Senescent stromal-derived osteopontin promotes preneoplastic cell growth. *Cancer Res*. **69**, 1230-1239.

Ren, J., Stroncek, D. F., Zhao, Y., Jin, P., Castiello, L., Civini, S., ... Sabatino, M. (2013). Intra-subject variability in human bone marrow stromal cell (BMSC) replicative senescence: molecular changes associated with BMSC senescence. *Stem Cell Res*. **11**, 1060-1073.

Ritchie, M. E., Phipson, B., Wu, D., Hu, Y., Law, C. W., Shi, W., & Smyth, G. K. (2015). limma powers differential expression analyses for RNA-sequencing and microarray studies. *Nucleic Acids Res*. **43**, e47.

Schwarze, S. R., DePrimo, S. E., Grabert, L. M., Fu, V. X., Brooks, J. D., & Jarrard, D. F. (2002). Novel pathways associated with bypassing cellular senescence in human prostate epithelial cells. *J Biol Chem*. **277**, 14877-14883.

Shah, P. P., Donahue, G., Otte, G. L., Capell, B. C., Nelson, D. M., Cao, K., ... Berger, S. L. (2013). Lamin B1 depletion in senescent cells triggers large-scale changes in gene expression and the chromatin landscape. *Genes Dev*. **27**, 1787-1799.

Yang, J., Huang, T., Petralia, F., Long, Q., Zhang, B., Argmann, C., ... Tu, Z. (2015). Synchronized age-related gene expression changes across multiple tissues in human and the link to complex diseases. *Sci Rep*. **5**, 15145.

Yu, G., Wang, L. G., Han, Y., & He, Q. Y. (2012). clusterProfiler: an R package for comparing biological themes among gene clusters. *OMICS*. **16**, 284-287.

Zhang, H., Pan, K. H., & Cohen, S. N. (2003). Senescence-specific gene expression fingerprints reveal cell-type-dependent physical clustering of up-regulated chromosomal loci. *Proc Natl Acad Sci U S A*. **100**, 3251-3256.
